# SARS‐CoV‐2 Infection Among Nursing Home Healthcare Workers: A Longitudinal Study in North‐Eastern Italy

**DOI:** 10.1111/irv.70056

**Published:** 2024-12-19

**Authors:** Valentina Rosolen, Diana Menis, Luigi Castriotta, Fabio Barbone, Francesca Larese Filon

**Affiliations:** ^1^ Central Directorate for Health, Social Policies and Disability Trieste Friuli Venezia Giulia Region Italy; ^2^ Department of Medicine University of Udine Udine Italy; ^3^ Institute of Hygiene and Evaluative Epidemiology Friuli Centrale Healthcare University Trust Udine Italy; ^4^ Department of Medicine, Surgery and Health Sciences University of Trieste Trieste Italy; ^5^ Occupational Medicine Unit Giuliano Isontina Healthcare University Trust Trieste Italy

**Keywords:** COVID‐19, health personnel, nursing homes, occupational health, SARS‐CoV‐2

## Abstract

**Background:**

During the pandemic, a surveillance program to monitor COVID‐19 infection among healthcare workers was established in Friuli Venezia Giulia Region (FVG), Italy. The aim of our study was to measure the risk of acquiring SARS‐CoV‐2 infection among nursing home employees by job title.

**Methods:**

From March 1, 2020, to March 31, 2023, a retrospective population‐based longitudinal study was conducted in 8880 nursing home employees. For each employee, all swabs up to the first positive result (*n* = 211.534) were considered. The study period was divided in six phases based on epidemic waves. Generalized estimated equations method for longitudinal binary data was applied with a time lag of a month, in each phase, obtaining an odds ratio (OR) and 95% confidence limit (95% CI) for each job category.

**Results:**

In Phase 1 (1.3.2020–30.6.2020), compared with administrative assistants, jobs with high patient contact were at increased risk of infection: The OR and 95% CI were 3.52 (1.44–8.56) and 2.96 (1.15–7.66) in healthcare elementary occupation and physicians/nurses, respectively. Corresponding associations in Phase 2 (1.7.2020–31.1.2021) were 1.54 (1.18–2.02) and 1.41 (1.04–1.91). On the contrary, in Phase 6 (20.12.2021–31.3.2023) physicians/nurses were at a decreased risk (0.73 [0.58–0.91]).

**Conclusions:**

In nursing homes, the risk of COVID‐19 infection varied by job title and pandemic phase. Virus higher infectivity, probability of closer contact, and better adherence to infection prevention control may explain part of these differences. Stronger nursing home–specific surveillance in patients and employees should be extended worldwide to control this high global burden of disease communities.

## 1 Introduction

Healthcare workers (HCWs) are at increased risk of developing COVID‐19 because of contact with patients and colleagues. To reduce such risk in Friuli Venezia Giulia Region (FVG) (Italy), a common protocol for periodical screening in nursing homes started in the early phase of the pandemic [1, 2, 3, 4, 5, 6]. All HCWs underwent nasopharyngeal swab tests for SARS‐CoV‐2 detection using real‐time PCR with (a) symptoms suggestive of COVID‐19, (b) close contact with positive cases, and (c) routinely monthly. This protocol allowed for a better control of the COVID‐19 pandemic and for establishing an efficient epidemiologic surveillance program within the regional, publicly employed, healthcare workforce. However, although information extracted from the surveillance program in hospitals was rather complete, information on workers employed in nursing homes was limited [7, 8, 9, 10, 11].

Before the COVID‐19 vaccination program was implemented in Italy on December 27, 2020, anecdotal information suggests that this subgroup of HCWs was at increased risk of developing COVID‐19 [12]. The anti‐COVID‐19 vaccination (mandated from April 1, 2021, for HCWs) led to almost complete vaccination coverage, ultimately protecting nursing home residents [13] and reducing absenteeism among a key occupational category during the pandemic [14]. Indeed, in hospitals, absenteeism among staff due to COVID‐19 reached values of around 50% [15]. These are unplanned absences often managed with extra shifts and consequently increased pressure from other staff. Consequently, healthcare workers are at risk of not only infection but also of physical, mental, and burnout overload.

This study aims to assess the association between the first SARS‐CoV‐2 infection and job title among nursing home employees in FVG.

## 2 Methods

### 2.1 Population

Regions in Italy were responsible during the pandemic for organizing and implementing vaccination and testing strategies at the local level, as well as collecting and reporting health surveillance data [16]. The study population included nursing home employees, residing and working in FVG between March 1, 2020, and March 31, 2023, who underwent at least one molecular or antigenic SARS‐CoV‐2 swab at a public or private, accredited facility, and aged 18 to 70 years at the date of their first swab. For each employee, all swabs, up to the first positive swab result, in the study period, with diagnostic validity for COVID‐19, according to the surveillance policy of regional and national authorities [17, 18] were considered. In detail, according to Italian regulations of the time and quality control procedures, the following swabs were excluded from the study: (a) swabs performed on employees not residing in FVG at the time of swab collection; (b) salivary molecular swabs taken before May 14, 2021; (c) antigenic swabs with a positive result taken before December 17, 2020; (d) antigenic swabs with a positive result taken in pharmacies before December 31, 2021; (e) all swabs after the first swab with a positive result; and (f) swabs whose result was a false positive.

### 2.2 Study Design and Data Source

We conducted a retrospective population‐based longitudinal study including all valid swabs, performed on employees of nursing homes, residing and working in FVG at the date of swab collection, to assess any differences according to the job they performed over the study period. The outcome of interest was the first positive swab for SARS‐CoV‐2 virus for each employee over the study period.

The data source was the regional health data warehouse managed by a company that performs data pseudonymization using an algorithm not known to the authors. Therefore, the authors of the present study were not aware of any identifying data attributable to the enrolled subjects. Information included HCWs demographics and job titles (alphanumeric string) and COVID‐19 testing results (positive or negative); race and ethnicity were not available. A team including an occupational physician, two hygienists, and a statistician interpreted job information and classified employees in the following broader job title categories: (i) healthcare elementary occupation (nutrition, hydration, washing, dressing, caring, moving, and generic handling of patients), (ii) physician (patients' anamnesis and examination, medications prescribing, and diagnostic test performing and interpreting), (iii) nurse (medications delivery, direct patients' care, patients monitoring, recording pulsing and temperature, etc.), (iv) other professional (social workers, educators, physical therapists, speech therapists, and psychologists), (v) support functions (laundry, meal preparation, equipment and building maintenance, hairdresser or pedicurist, doorman, and cleaning attendant), and (vi) administrative assistant.

### 2.3 Statistical Analyses

Gender, age, and province of residence in FVG at the date of collection of the first swab performed in the study period of each nursing home employee were considered as covariates.

To account for the different epidemic waves, also sustained by different SARS‐CoV‐2 variants, the study period was divided into the following phases [19]: (i) Phase 1 (1.3.2020–30.6.2020) characterized by the first pandemic peak and the national lockdown; (ii) Phase 2 (1.7.2020–31.1.2021) characterized by the second pandemic peak and the introduction of the vaccination campaign against COVID‐19; (iii) Phase 3 (1.2.2021–17.5.2021) characterized by the prevalence of the Alpha variant; (iv) Phase 4 (18.5.2021–14.10.2021) characterized by the prevalence of the Delta variant and before the law of 15.10.2021 was approved which obliged all workers to have the anti‐COVID‐19 vaccination certificate or to have carried out a recent (within 48 h) swab to test for the SARS‐CoV‐2 virus with negative results; (v) Phase 5 (15.10.2021–19.12.2021) characterized by the prevalence of the Delta variant and the approval of the law of 15.10.2021; (vi) Phase 6 (20.12.2021–31.3.2023) characterized by the prevalence of the Omicron variant and related subvariants.

The main characteristics of nursing home employees are presented as frequency and percentage distributions. To assess whether during the study period the number of swabs performed varied by job title, the mean number of swabs taken per specific job by each month and year of swab collection was calculated. The cumulative percentage of the first dose of anti‐COVID‐19 vaccination carried out by each nursing home employee was computed by month and year of vaccine administration and job title, to describe the progression of the vaccination coverage among the nursing home employees over the study period.

To account for the repeated swabs performed by each employee and for variations in COVID‐19 incidence rates, Generalized Estimated Equations (GEE) method for longitudinal binary data was applied in each of the six phases. GEE estimating marginal models studied the effect of the different exposures among nursing home employees on first positivity to SARS‐CoV‐2 virus swabs over time in each phase. The GEE coefficient allows measuring the Odds Ratio (OR) and the related 95% confidence interval (95% CI) that may be interpreted as the association between COVID‐19 infection and a job title that entailed a higher occupational exposure to SARS‐CoV‐2 contact (i.e., healthcare elementary occupations, physicians, nurses, workers with support functions, and other professionals) versus a job title at the lowest exposure level, thus, the reference category was identified as nursing home administrative assistants. To apply the GEE models, a time lag of a month was considered and in situations where multiple swabs were collected from the same employee in a single time lag, the following was considered: (1) the swab with the first positive result or (2) in case of persistent negative results, the first swab in chronological order. Then, for each month and year, the number of employees, with a first positive swab and the number of employees persistently negative were counted. GEE models were applied in each phase including the following variables: job performed by each employee, month and year, gender, age, and province of residence of the employee. Because the number of physicians and nurses in most phases was sparse, these jobs were aggregated to estimate ORs and 95% CIs.

For each of the six phases, the frequency and percentage distributions of the first positive swabs (considered in GEE models) were calculated for the main characteristics of the employees who performed at least one swab in the phase.

The SAS software (version 9.4 SAS Institute Inc., Cary, NC, USA) was used for all statistical elaborations.

## 3 Results

**FIGURE 1:**
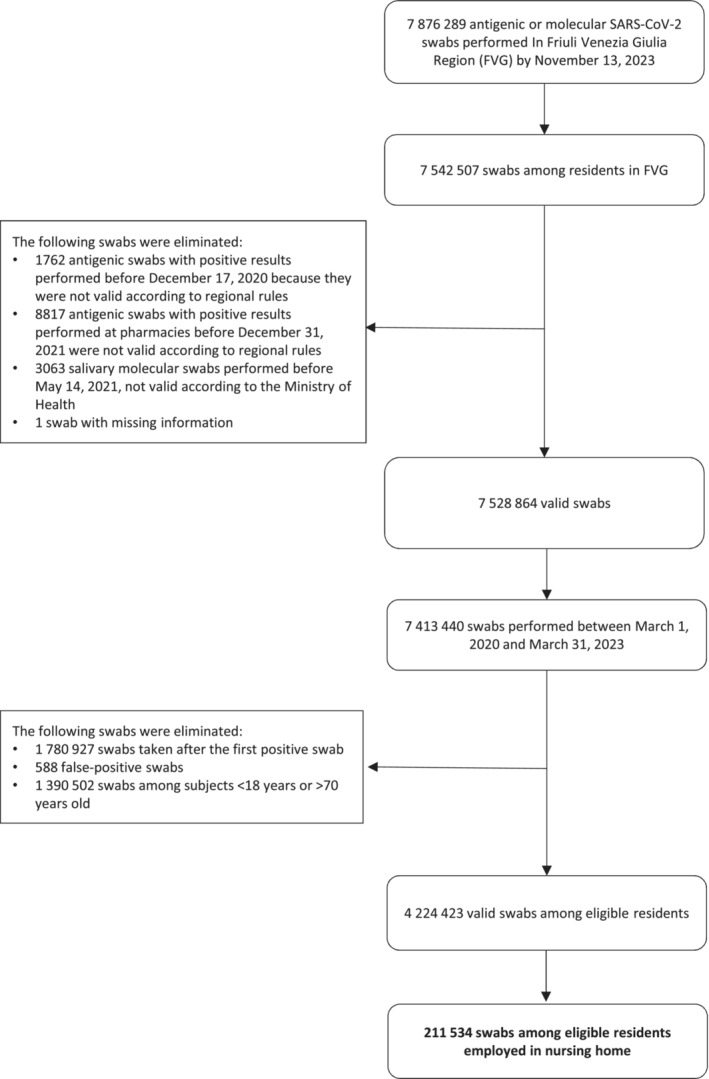
Flow diagram of SARS‐CoV‐2 testing in the population base.

A flow diagram of valid SARS‐CoV‐2 swabs is reported in Figure 1. The study population included 8880 nursing home staff who underwent, over the study period, a total of 211.534 valid swabs before testing positive, that is, an average of 23.8 swabs per employee. As presented in Table 1, among nursing home staff, the cumulative incidence of COVID‐19 infection was 69.5%. Approximately 70.0% of them were younger than 50 years old; 84.4% were female; 52.2% worked in a healthcare elementary occupation, less than 10.0% were either a physician or a nurse, 25.9% were employed in support functions, whereas the remaining participants were administrative assistants or other professionals.

**TABLE 1:** Main characteristics of the 8880 nursing home employees considered in the study period.

Main characteristics of nursing home employees	
First infection of COVID‐19	*N* (%)
No	2710 (30.5)
Yes	6170 (69.5)
**Age** ^**a**^ **of employee**	
18–30	1393 (15.7)
31–40	1749 (19.7)
41–50	2597 (29.3)
51–60	2578 (29.0)
61–70	563 (6.3)
**Gender of employee**	
Female	7496 (84.4)
Male	1384 (15.6)
**Province of residence of employee** ^**b**^	
Gorizia	982 (11.1)
Pordenone	1952 (22.0)
Trieste	2070 (23.3)
Udine	3876 (43.7)
**Job**	
Healthcare elementary occupation	4647 (52.5)
Physician	42 (0.5)
Nurse	833 (9.4)
Other professional^c^	576 (6.5)
Worker with support functions^d^	2294 (25.9)
Administrative assistant	468 (5.3)

^a^ Age, in years, computed at the date of collection of the first swab performed during the study period.

^b^ Province of residence at the date of collection of the first swab performed during the study period.

^c^ Social worker, educator or entertainer, physical therapist, speech therapist, and psychologist.

^d^ Laundry, meal preparation, maintenance, hairdresser or pedicurist, doorman, and cleaning attendant.

Table S1 displays the frequency distribution of tests performed and those used in the GEE models by each phase. As described in the material and methods section, to apply the GEE models a time lag of a month was considered, so for each time lag the number of employees with a first positive swab and the number of employees persistently negative were counted. Table S2 reports the frequency and percentage tests' distributions by main employee's characteristics and phase. In Figures S1–S6, the mean number of tests per employee is presented according to job title and calendar month and year: the mean number of tests per employee was very similar both among job title and calendar month and year during Phase 1 (from 1.2 to 1.6), Phase 3 (from 2.5 to 3.2), Phase 4 (from 2.0 to 2.5), and Phase 5 (from 2.5 to 3.4); during Phase 2 and Phase 6, the mean within each calendar month and year was very similar across job title.

**FIGURE 2:**
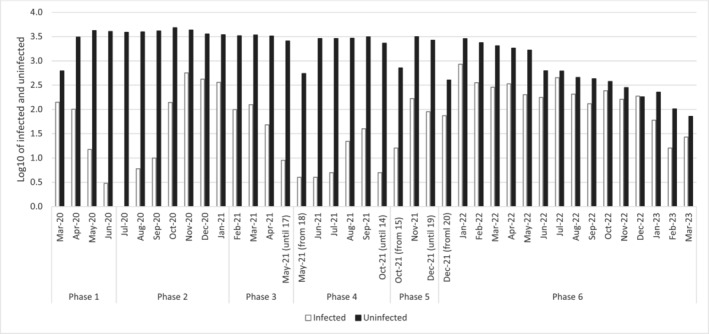
COVID‐19 positive and negative tests among nursing home employees by phase, month, and year. Phase 1: from March 1, 2020, to June 6, 2020, characterized by the first pandemic peak and national lockdown. Phase 2: from July 1, 2020, to January 31, 2021, characterized by the second pandemic peak and, on December 27, 2020, start of COVID‐19 vaccination. Phase 3: from February 1, 2021, to May 17, 2021, characterized by prevalent circulation of the Alpha variant. Phase 4: from May 18, 2021, to October 14, 2021, characterized by prevalent circulation of the Delta variant. Phase 5: from October 15, 2021, to December 19, 2021, characterized by prevalent circulation of the Delta variant and the introduction of the Green Pass Policy. Phase 6: from December 20, 2021, to March 31, 2023, characterized by prevalent circulation of the Omicron variant.

**FIGURE 3:**
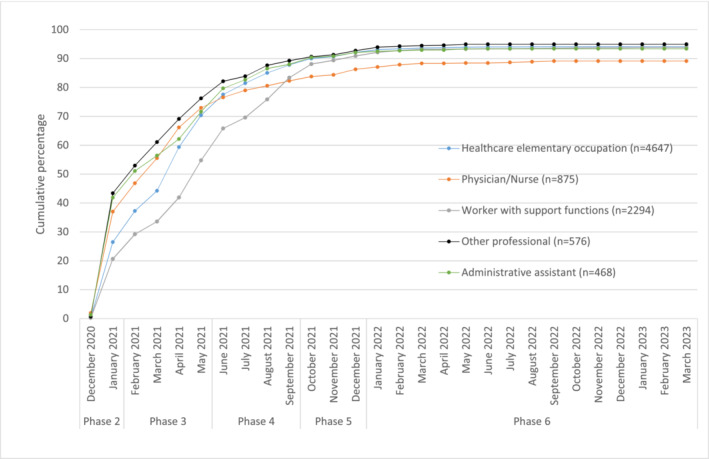
COVID‐19 vaccination coverage (first dose) among nursing home employees.

Figure 2 shows the distribution of the number of infected and uninfected nursing home employees by phase, month, and year. The dynamic of first dose of anti‐COVID‐19 vaccination coverage among nursing home staff is shown in Figure 3.

**FIGURE 4:**
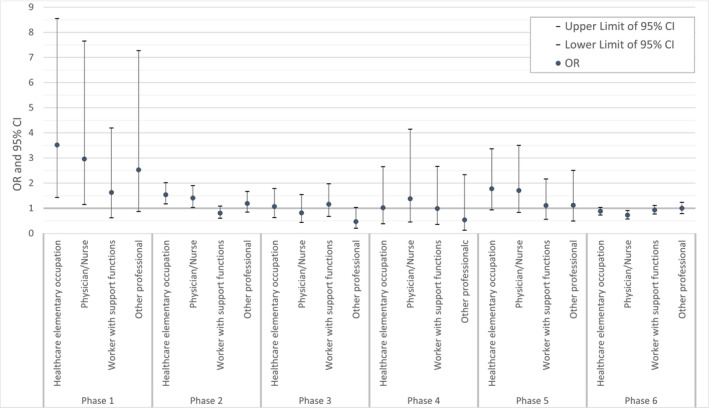
Associations between job title and first SARS‐CoV‐2 virus infection, by phase. Odds ratio (OR) and 95% confidence interval (95% CI) from GEE models adjusted for time lag, gender, age group, and province of residence. Reference category: nursing home administrative assistants.

The associations between job title and first SARS‐CoV‐2 virus infection by phase are presented in Figure 4 and Table S3, according to GEE models adjusted for month and year, job, gender, age groups, and province of residence. During Phase 1, compared with administrative assistants, healthcare elementary occupations (OR = 3.52; 95% CI = 1.44–8.56) and physicians/nurses (OR = 2.96; 95% CI = 1.15–7.66) were exposed to a higher risk to be infected. Again, in Phase 2, healthcare elementary occupations (OR = 1.54; 95% CI = 1.18–2.02) and physicians/nurses (OR = 1.41; 95% CI = 1.04–1.91) showed a higher probability of infection, although such an excess risk is less evident than in Phase 1. In Phase 3, 4, and 5, no clear associations emerged between job title and probability of infection. Finally, during Phase 6, physicians/nurses (OR = 0.73; 95% CI = 0.58–0.91) showed a lower probability of infection than administrative assistants.

## 4 Discussion

Compared with other settings, because of the disproportioned number of deaths among residents, nursing homes have been defined as the “ground zero” of the COVID‐19 pandemic [20]. Many factors could have contributed to perpetuating the viral transmission in these facilities: residents' low immunocompetence and high comorbidity, close contact among residents and HCWs, staff shortage, inappropriate use of personal protective equipment (PPE), and insufficient testing or shortage of masks/gloves [21].

Reports transmitted by the Regional Task Force to the Italian National Institute of Health and the National Ministry of Health weekly demonstrate that outbreaks were present in nursing homes during 143 out of the 165 weeks included in the study period [22].

The region of Friuli‐Venezia Giulia, in north‐eastern Italy, has a population of 1,195,630 inhabitants and an area of 7845 km^2^. The population of this region is significantly older than the rest of the Italian population, and it is predominantly a rural territory composed of mountains and hills, divided in four provinces with four regional capitals: Pordenone (population density 137.4 inhabitants per km^2^), Udine (population density 105.9 inhabitants per km^2^), Gorizia (population density 298.0 inhabitants per km^2^) and Trieste (population density 1097.7 inhabitants per km^2^).

Residential facilities for the elderly are distributed over the territory as follows: 70 facilities in the province of Trieste, 53 in the province of Udine, 27 in the province of Pordenone, and 15 in the province of Gorizia.

In our study, we included exclusively nursing homes facilities. These facilities, in our region, offer a type of permanent residence to users with different degrees of autonomy and intensity of care. Nevertheless, in all facilities, isolation of users with confirmed disease was adopted and operators were trained on the appropriate use of personal protective equipment (PPE) in suspected or confirmed cases. Visitor access was initially banned and later modulated according to the epidemiological trend of infections. In addition, new admissions of residents to social care residential facilities were restricted and only took place following a health assessment and a negative swab. There were not COVID‐designated nursing homes during the pandemic. However, the specific indications in this and other settings have changed over the course of the different phases of the pandemic.

Therefore, given the specificity of each phase of the COVID‐19 pandemic, the results of this study will be discussed by phase.

### 4.1 Phase 1 (March 1, 2020, to June 30, 2020)

During the first pandemic peak, physicians/nurses had almost a threefold higher risk of infection (OR = 2.96; 95% CI = 1.15–7.66) and healthcare elementary occupations had more than 3 times the risk of infection (OR = 3.52; 95% CI = 1.44–8.56), compared with administrative assistants. In FVG, access to nursing homes by visitors was prohibited, except in exceptional cases. Standard preventive guidelines dealt with asymptomatic, suspected, or confirmed positive residents [23]. HCWs in nursing homes were required to carry out swabs every 7–15 days [24] and every 48–72 h in case of close contact [25]. Even if not specified in the procedures, administrative workers were also being tested periodically as HCWs. From March 8, 2020, to May 18, 2020, the entire country experienced a compulsory lockdown and during the limited times when subjects were allowed to exit their home, face masking was mandatory in all indoor and outdoor public places. Taking into account outbreaks mapping, nursing homes and some hospital wards were the settings where the virus circulated most. Additionally, a study conducted in our region showed a tendency to manage COVID‐19‐positive residents directly within nursing homes instead of hospitalizing them [26].

In this phase, published data in nursing homes and long‐term care facilities are mixed. Some authors observed that the prevalence of infection was higher in staff with versus without patient contact [27, 28], whereas other authors did not observe differences between these categories [29]. Ladhani et al. observed a higher prevalence of positivity among permanent staff who had regular contact with residents and worked in multiple care homes (3.0‐fold higher risk; *p* < 0.001) compared with staff working in a single care home [29]. In this regard external evidence is mixed [30, 31], and unfortunately, this information is not available for our population.

### 4.2 Phase 2 (July 1, 2020, to January 31, 2021)

In Phase 2, healthcare elementary occupations (OR = 1.54; 95% CI = 1.18–2.02) and physicians/nurses (OR = 1.41; 95% CI = 1.04–1.91) showed a higher probability of infection compared with administrative staff although the difference in terms of risk was smaller compared with Phase 1. During this period, access by visitors was defined by clean paths, with major restrictions in case of a facility outbreak and the Infection Prevention and Control (IPC) procedures and frequency of testing corresponded to Phase 1 [32]. The literature supports the evidence that HCWs without direct contact with residents had a lower risk of infection compared with HCWs with direct contact [21, 31, 33].

### 4.3 Phases 3,4,5 (February 1, 2021, to December 19, 2021)

In our study, during these phases, no increased risk was found for HCWs compared with clerks. The Ministry of Health removed nursing home restrictions to visitors' access if they presented proof of complete vaccination or a recent SARS‐CoV‐2 negative test (i.e., Green Pass) [34].

On the contrary, the results from unadjusted analyses of a study from Quebec and a study from France show a higher risk of infection for HCWs in contact with patients [35, 36]. Both studies did not separate hospital HCWs from those employed in nursing homes. Peculiarly, in the Canadian study, the highest risk was found for housekeeping staff (aOR, 3.6) rather than for patient‐support assistants (aOR, 1.9) or nursing staff (aOR, 1.4). These results suggested a potential risk associated with waste management [35] possibly due to a lack of IPC training and a low risk perception of this category.

### 4.4 Phase 6 (December 20, 2021, to March 31, 2023)

Physicians and nurses (OR = 0.73; 95% CI = 0.58–0.91) showed a lower probability of infection than administrative assistants. In February 2022, the Regional Authority recommended a periodic screening swab every 10 days for workers in contact with patients [37].

During this phase, when PPE was easily available and the population could move freely, viral transmission was again predominantly in the community. Therefore, it is also possible that the category of HCWs with higher contact with patients but also a better risk perception was able to avoid more effectively community transmission. Unfortunately, we were unable to trace literature data in this phase.

The fact that studies in the hospital setting [38, 39, 40, 41, 42] conclude that there are no differences in infection risk depending on professional role and department along all the study period, makes us reflect on the importance of IPC in both settings. Indeed, although nursing home patients are generally less self‐sufficient, requiring more direct care that increases the risk of infection transmission, a need to improve IPC procedures in this setting, has been observed [43, 44]. In fact, it is likely that HCWs are better trained within the hospital setting than within the territory. Literature supports the idea that PPE and routine swab screening are effective strategies to reduce the absenteeism of HCWs during the COVID‐19 pandemic [45, 46].

A strength of our study is the long observation period compared with the literature (equal to or shorter than 6 months) [21, 27, 31, 33, 35, 36] and the complete, population‐based availability of data on both swab testing and vaccination coverage among workers. One limitation is the lack of data on the mobility of workers between different nursing homes, which according to the literature may increase the risk of infection.

## 5 Conclusions

We observed that workplace conditions influence the risk of transmission in nursing homes. During the first two phases of the pandemic, we observed an increased risk of infection in the categories with greater contact with patients. The fact that these findings are not observed in the hospital setting supports the hypothesis that IPC procedures can mitigate the risk. On the other hand, in the later stages of the pandemic, when the virus was transmitted freely within the community, physicians and nurses, probably more prepared than other employees, presented a lower risk of infection.

In the future, a better understanding of the risk of infection among staff categories may identify additional personalized interventions to protect vulnerable residents in nursing homes, avoid days of absence, and indirectly reduce the mental health effects on healthcare workers.

## Author Contributions

**Valentina Rosolen:** formal analysis (lead), methodology (equal), visualization (equal), writing – original draft preparation (equal), software (lead). **Diana** **Menis:** visualization (equal), writing – original draft preparation (equal). **Luigi** **Castriotta:** methodology (equal), writing – review and editing. **Fabio** **Barbone:** conceptualization (equal), methodology (equal), writing – original draft preparation (equal), writing – review and editing. **Francesca Larese Filon:** conceptualization (equal), writing – review and editing.

## Ethics Statement

The local Ethic Committee (CEUR) approved the study on April 1, 2021, n.032_2021H.

## Consent

The authors had access only to pseudonymized data at origin. Because the identities of the subjects involved in the study were unknown, informed consent could not be obtained.

## Conflicts of Interest

The authors declare no conflicts of interest.

### Peer Review

The peer review history for this article is available at https://www.webofscience.com/api/gateway/wos/peer‐review/10.1111/irv.70056.

## Supporting information


**Table S1.** Frequency distribution of the swab performed and the swab used in GEE models taking into account the time lag, during each phase
**Figure S1.** Mean of swab performed by employee ’ s jobs in the month and year of swab collection date, during Phase 1
**Figure S2.** Mean of swab performed by employee ’ s jobs in the month and year of swab collection date, during Phase 2
**Figure S3.** Mean of swab performed by employee ’ s jobs in the month and year of swab collection date, during Phase 3
**Figure S4.** Mean of swab performed by employee ’ s jobs in the month and year of swab collection date, during Phase 4
**Figure S5.** Mean of swab performed by employee ’ s jobs in the month and year of swab collection date, during Phase 5
**Figure S6.** Mean of swab performed by employee ’ s jobs in the month and year of swab collection date, during Phase 6
**Table S2.** Frequency and percentage distribution of swabs with first positive results and negative results by the main characteristics of the employees who performed at least one swab during each phase
**Table S3.** Odds ratio (OR) and 95% confidence interval (95% CI) of multiple GEE models, by phase

## Data Availability

The data underlying this article will be shared, in aggregate form, on reasonable request to the corresponding author.
