# Supplementary material for: SARS‐CoV‐2 Infection Among Nursing Home Healthcare Workers: A Longitudinal Study in North‐Eastern Italy
Source: Influenza Other Respir Viruses. 2024 Dec 19;18(12):e70056. doi: 10.1111/irv.70056 (PMC11658915; doi:10.1111/irv.70056)
Supplement: Supplementary file 1 — Table S1. Frequency distribution of the swab performed and the swab used in GEE models taking into account the time lag, during each phase Figure S1. Mean of swab performed by employee ’ s jobs in the month and year of swab collection date, during Phase 1 Figure S2. Mean of swab performed by employee ’ s jobs in the month and year of swab collection date, during Phase 2 Figure S3. Mean of swab performed by employee ’ s jobs in the month and year of swab collection date, during Phase 3 Figure S4. Mean of swab performed by employee ’ s jobs in the month and year of swab collection date, during Phase 4 Figure S5. Mean of swab performed by employee ’ s jobs in the month and year of swab collection date, during Phase 5 Figure S6. Mean of swab performed by employee ’ s jobs in the month and year of swab collection date, during Phase 6 Table S2. Frequency and percentage distribution of swabs with first positive results and negative results by the main characteristics of the employees who performed at least one swab during each phase Table S3. Odds ratio (OR) and 95% confidence interval (95% CI) of multiple GEE models, by phase [file IRV-18-e70056-s001.docx]

**Table S1. Frequency distribution of the swab performed and the swab used in GEE models taking into account the time lag, during each phase**

| **Phases** | **N° of swabs performed** | **N° of swabs used in GEE models** |
| --- | --- | --- |
| **Phase 1** | 17902 | 12192 |
| **Phase 2** | 57707 | 29671 |
| **Phase 3** | 34881 | 12792 |
| **Phase 4** | 33746 | 16651 |
| **Phase 5** | 19762 | 8519 |
| **Phase 6** | 47536 | 20299 |

**Table S2. Frequency and percentage distribution of swabs with first positive results and negative results by the main characteristics of the employees who performed at least one swab during each phase**

| **Covariates** | **Phase 1** | | **Phase 2** | | **Phase 3** | | **Phase 4** | | **Phase 5** | | **Phase 6** | |
| --- | --- | --- | --- | --- | --- | --- | --- | --- | --- | --- | --- | --- |
|  | **N° positive result swabs** | **N° negative result swabs** | **N° positive result swabs** | **N° negative result swabs** | **N° positive result swabs** | **N° negative result swabs** | **N° positive result swabs** | **N° negative result swabs** | **N° positive result swabs** | **N° negative result swabs** | **N° positive result swabs** | **N° negative result swabs** |
| **Job, n (%)** |  |  |  |  |  |  |  |  |  |  |  |  |
| Healthcare elementary occupation | 167 (64.5) | 6351 (53.4) | 918 (61.1) | 14609 (52.0) | 147 (52.7) | 6160 (49.4) | 41 (51.9) | 8168 (49.4) | 161 (58.8) | 4003 (48.7) | 1930 (51.3) | 8236 (50.0) |
| Physician/Nurse | 35 (13.5) | 1456 (12.2) | 190 (12.7) | 3230 (11.5) | 26 (9.3) | 1342 (10.8) | 10 (12.7) | 1693 (10.2) | 31 (11.3) | 833 (10.1) | 319 (8.5) | 1706 (10.4) |
| Worker with support functions^a^ | 38 (14.7) | 2723 (22.9) | 232 (15.5) | 6691 (23.8) | 78 (28.0) | 3235 (25.9) | 20 (15.3) | 4365 (26.4) | 56 (20.4) | 2259 (27.5) | 1007 (26.8) | 4372 (26.5) |
| Other professional^b^ | 14 (5.4) | 748 (6.3) | 96 (6.4) | 1943 (6.9) | 11 (3.9) | 942 (7.6) | 3 (3.8) | 1247 (7.6) | 15 (5.5) | 623 (7.6) | 287 (7.6) | 1181 (7.2) |
| Administrative assistant | 5 (1.9) | 613 (5.2) | 66 (4.4) | 1621 (5.8) | 17 (6.1) | 801 (6.4) | 5 (6.3) | 1053 (6.4) | 11 (4.0) | 511 (6.2) | 222 (5.9) | 995 (6.0) |
| **Age^c^ , n (%)** |  |  |  |  |  |  |  |  |  |  |  |  |
| 18-30 | 24 (9.2) | 1355 (11.4) | 230 (15.3) | 3876 (13.8) | 52 (18.5) | 1805 (14.4) | 6 (7.5) | 2391 (14.4) | 39 (14.2) | 1205 (14.6) | 578 (15.3) | 2332 (14.1) |
| 31-40 | 44 (16.9) | 2088 (17.5) | 269 (17.9) | 4831 (17.2) | 57 (20.3) | 2182 (17.4) | 29 (36.3) | 2917 (17.6) | 58 (21.2) | 1511 (18.3) | 767 (20.3) | 2948 (17.8) |
| 41-50 | 80 (30.8) | 3874 (32.5) | 452 (30.1) | 8714 (30.9) | 91 (32.4) | 3855 (30.8) | 20 (25.0) | 5033 (30.4) | 104 (38.0) | 2419 (29.3) | 1057 (28.0) | 4610 (27.9) |
| 51-60 | 96 (36.9) | 3932 (33.0) | 463 (30.8) | 9066 (32.2) | 67 (23.8) | 3910 (31.2) | 22 (27.5) | 5160 (31.1) | 57 (20.8) | 2544 (30.9) | 1094 (29.0) | 5394 (32.6) |
| 61-70 | 16 (6.2) | 683 (5.7) | 90 (6.0) | 1680 (6.0) | 14 (5.0) | 764 (6.1) | 3 (3.8) | 1070 (6.5) | 16 (5.8) | 566 (6.9) | 275 (7.3) | 1244 (7.5) |
| **Gender, n (%)** |  |  |  |  |  |  |  |  |  |  |  |  |
| Female | 222 (85.4) | 10243 (85.8) | 1284 (85.4) | 23764 (84.4) | 244 (86.8) | 10455 (83.5) | 67 (83.8) | 13783 (83.2) | 222 (81.0) | 6858 (83.2) | 3204 (85.0) | 13849 (83.8) |
| Male | 38 (14.6) | 1689 (14.2) | 220 (14.6) | 4403 (15.6) | 37 (13.2) | 2061 ( 16.5) | 13 (16.3) | 2788 (16.8) | 52 (19.0) | 1387 (16.8) | 567 (15.0) | 2679 (16.2) |
| **Province of residence^d^, n (%)** |  |  |  |  |  |  |  |  |  |  |  |  |
| Gorizia | 13 (5.0) | 1521 (12.8) | 152 (10.1) | 3133 (11.1) | 39 (13.9) | 1996 (16.0) | 10 (12.5) | 2863 (17.3) | 43 (15.7) | 1373 (16.7) | 428 (11.4) | 2220 (13.4) |
| Pordenone | 23 (8.9) | 1835 (15.4) | 258 (17.2) | 3243 (11.5) | 35 (12.5) | 846 (6.8) | 12 (15.0) | 845 (5.10) | 58 (21.2) | 443 (5.4) | 912 (24.2) | 1056 (6.4) |
| Trieste | 153 (58.9) | 2094 (17.6) | 325 (21.6) | 6807 (24.2) | 63 (22.4) | 3734 (29.8) | 40 (50.0) | 5159 (31.1) | 111 (40.5) | 2504 (30.4) | 747(19.8) | 4018 (24.3) |
| Udine | 71 (27.3) | 6482 (54.3) | 769 (51.1) | 14984 (53.2) | 144 (51.3) | 5940 (47.5) | 18 (22.5) | 7704 (46.5) | 62 (22.6) | 3925 (47.6) | 1684 (44.7) | 9234 (55.9) |
| ^a^Jobs considered: laundry attendant, meal preparation attendant, maintenance attendant, hairdresser or pedicurist, doorman, and cleaning attendant. ^b^Jobs considered: social worker, educator or entertainer, physical therapist, speech therapist, and psychologist. ^c^Age, in years, computed at the date of collection of the first swab performed during the study period. ^d^Province of residence at the date of collection of the first swab performed during the study period. | | | | | | | | | | | | |

**Figure S1. Mean of swab performed by employee’s jobs in the month and year of swab collection date, during Phase 1**

**Figure S2. Mean of swab performed by employee’s jobs in the month and year of swab collection date, during Phase 2**

**Figure S3. Mean of swab performed by employee’s jobs in the month and year of swab collection date, during Phase 3**

**Figure S4. Mean of swab performed by employee’s jobs in the month and year of swab collection date, during Phase 4**

**Figure S5. Mean of swab performed by employee’s jobs in the month and year of swab collection date, during Phase 5**

**Figure S6. Mean of swab performed by employee’s jobs in the month and year of swab collection date, during Phase 6**

**Table S3. Odds ratio (OR) and 95% confidence interval (95% CI) of multiple GEE models, by phase**

|  | **Phase 1** | | **Phase 2** | | **Phase 3** | | **Phase 4** | | **Phase 5** | | **Phase 6** | | |
| --- | --- | --- | --- | --- | --- | --- | --- | --- | --- | --- | --- | --- | --- |
| **Covariates** | **OR^a^** | **95% CI** | **OR^a^** | **95% CI** | **OR^a^** | **95% CI** | **OR^a^** | **95% CI** | **OR^a^** | **95% CI** | **OR^a^** | **95% CI** |  |
| **Job, n (%)** |  |  |  |  |  |  |  |  |  |  |  |  |  |
| Healthcare elementary occupation | 3.52 | 1.44 – 8.56 | 1.54 | 1.18 – 2.02 | 1.07 | 0.64 – 1.79 | 1.02 | 0.39 – 2.66 | 1.78 | 0.94 – 3.37 | 0.88 | 0.74 – 1.04 |  |
| Physician/Nurse | 2.96 | 1.15 – 7.66 | 1.41 | 1.04 – 1.91 | 0.82 | 0.44 – 1.55 | 1.38 | 0.46 – 4.15 | 1.71 | 0.84 – 3.51 | 0.73 | 0.58 - 0.91 |  |
| Worker with support functions^b^ | 1.63 | 0.63 – 4.20 | 0.81 | 0.61 – 1.09 | 1.16 | 0.68 – 1.98 | 0.99 | 0.36 – 2.67 | 1.11 | 0.57 – 2.17 | 0.93 | 0.78 – 1.12 |  |
| Other professionals^c^ | 2.53 | 0.88 – 7.28 | 1.19 | 0.85 – 1.68 | 0.47 | 0.21 – 1.04 | 0.54 | 0.13 – 2.34 | 1.12 | 0.50 – 2.51 | 1.00 | 0.80 – 1.24 |  |
| Administrative assistant | 1 | 1 | 1 | 1 | 1 | 1 | 1 | 1 | 1 | 1 | 1 | 1 |  |

^a^Adjusted for: time lag, job, gender, age groups and province of residence. ^b^Jobs considered: laundry attendant, meal preparation attendant, maintenance attendant, hairdresser or pedicurist, doorman, and cleaning attendant. ^c^Jobs considered: social worker, educator or entertainer, physical therapist, speech therapist, and psychologist.
